# Application of dynamic hydrogels for dental pulp regeneration: a comparative study on cellular behavior and inflammatory modulation

**DOI:** 10.3389/fdmed.2026.1847615

**Published:** 2026-08-12

**Authors:** Yara AlMaimouni, Weihao Yuan, Shorouq Houtari, Alireza Moshaverinia

**Affiliations:** 1 Department of Restorative Dental Sciences, College of Dentistry, Imam Abdulrahman Bin Faisal University, Dammam, Saudi Arabia; 2 School of Dentistry, University of California, Los Angeles, Los Angeles, CA, United States; 3 Department of Restorative Sciences, College of Dentistry, Kuwait University, Safat, Kuwait

**Keywords:** dynamic hydrogels, regenerative endodontics, dental pulp stem cells, immunomodulation, tissue engineering, dental biomaterials

## Abstract

**Introduction:**

Regenerative endodontic therapy aims to restore the pulp-dentin complex through biological approaches; however, current cell-free techniques often fail to achieve true dental pulp regeneration and may result in fibrous tissue formation or canal calcification. This study investigated dynamic hydrogels as advanced scaffolds for dental pulp stem cell (DPSC)-mediated regeneration, leveraging their viscoelastic properties to regulate cellular behavior and immune responses.

**Methods:**

Dynamic hydrogels were synthesized using host-guest chemistry (Gel-Mal/HA-Ada-CD-SH) to mimic aspects of the native extracellular matrix and were compared with non-dynamic Gel-Mal hydrogels. DPSCs and macrophages were encapsulated within the hydrogels to evaluate cellular viability, morphology, inflammatory marker expression, and gene expression profiles.

**Results:**

Dynamic hydrogels exhibited a distinct porous microarchitecture and significantly increased NF-κB and VEGF expression while reducing IL-1RA expression compared with non-dynamic hydrogels. Gene expression analysis demonstrated significantly increased TGFβ1, SMAD3, and MAPK expression, together with reduced CD80 expression, in macrophages cultured within dynamic hydrogels. Although iNOS expression was lower in the dynamic hydrogel group, the difference was not statistically significant.

**Discussion:**

These findings suggest that dynamic hydrogels do not simply suppress inflammatory signaling but may modulate inflammatory and tissue remodeling pathways while promoting angiogenesis-associated responses. Limitations of the study include the short-term in vitro design and evaluation of a limited panel of inflammatory and fibrosis-associated markers. Within these limitations, dynamic hydrogels demonstrated favorable cellular and immunomodulatory responses and may provide a biologically active microenvironment that supports cellular activities relevant to regenerative endodontic applications. Further in vivo studies are required to validate their translational potential.

## 1 Introduction

Regenerative endodontics is a biologically based procedure designed to physiologically replace damaged tooth structures, including dentin, pulp tissues, and cells of the pulp-dentin complex (1). This has interchangeable terminologies in literature, such as pulp revascularization, revitalization, and guided endodontic repair, but “regenerative endodontics” has become the most widely accepted term, reflecting its biological foundation.

Regenerative endodontic procedures can be achieved by two approaches including cell-free and cell-based therapies. Cell-free approaches rely on host-derived materials, such as blood clots, platelet-rich plasma (PRP), platelet-rich fibrin (PRF), and exosomes, often combined with growth factors, chemicals, and scaffolds. While these methods have high success rates evidenced by resolution of symptom and periapical healing, the canals are frequently filled with periapical tissues such as cementum, bone, periodontal ligament (PDL), and fibrous tissue, with high rates of canal calcification and obliteration reported (2, 3). In contrast, cell-based therapies use stem cells from autologous, allogeneic, or xenogeneic sources combined with growth factors and scaffolds, to promote complete regeneration of the pulp-dentin complex (4). A pilot study demonstrated a complete pulp regeneration in humans using stem cell-mediated techniques, marking a significant breakthrough in the field (4).

The success of regenerative endodontics is heavily dependent on the use of biomaterials, which serve as scaffolds to support cell attachment, proliferation, and differentiation, ultimately facilitating tissue regeneration (5, 6). A wide range of biomaterials has been studied in both *in vivo* and *in vitro* studies. *In vivo*, scaffolds such as blood clots, gel foam, collagen, platelet-rich plasma (PRP), platelet-rich fibrin (PRF), plasma rich in growth factors, and hydrogels such as gelatin, poly-L-lactic acid, self-assembly nanopeptides, oxidized alginate fibrin that have been studied (7, 8). *In vitro*, researchers have investigated decellularized extracellular matrix, magnetic nanofibers, hydrogels such as carboxymethyl cellulose-hydroxyapatite, tunable polyethylene glycol fibronectin, gelatin methacryloyl, and synthetic polymer-based hydrogels (9, 10).

Among these, hydrogels have emerged as particularly promising due to their biocompatibility and ability to mimic the natural extracellular matrix (ECM). For instance, a study demonstrated the controlled release of fibroblast growth factor-2 (FGF-2) from gelatin hydrogels, which promoted dentinal bridge formation in regenerated dental pulp (7). Similarly, another study highlighted the ability of hydrogel scaffolds to maintain cell viability and support the differentiation of dental pulp stem cells (8).

Moreover, hydrogels provide a supportive microenvironment that facilitates cell survival, proliferation, cell–matrix interactions, and tissue regeneration (11). Both non-dynamic (ND) and dynamic (D) hydrogels play critical roles in this process. ND-hydrogels provide a stable structural support and stability during tissue regeneration. On the other hand, D-hydrogels are advanced biomaterials characterized by reversible crosslinking networks that enable tunable mechanical properties, self-healing capabilities, and dynamic responsiveness to physiological stimuli. D-hydrogels provide a degradable and adaptable scaffold, which during the regenerative process ensures that they do not remain indefinitely (12, 13). Conversely, non-dynamic hydrogels rely primarily on permanent covalent crosslinks, resulting in more limited stress-relaxation behavior and network remodeling compared with dynamic hydrogels (14). Dynamic hydrogels have been reported to promote cell spreading and mechanotransduction-related responses, including enhanced cellular elongation and regulation of TGFβ/SMAD-associated signaling pathways (15, 16).

Innovations in biomaterials, such as viscoelastic hydrogels with dynamic crosslinks, are gaining attention for their potential in dental pulp disease treatment. These hydrogels can potentially mimic the natural extracellular matrix (ECM) with their adaptability to pH, temperature, and enzymatic activity which allows for control over the release of therapeutic agents (5, 7, 8). Such developments are particularly promising as they may improve the regenerative potential of current therapies.

Despite the significant advancements in hydrogel-based regenerative strategies, a critical research gap remains in understanding how the mechanical properties of hydrogels influence dental pulp stem cell behavior and their effect on immune response. Understanding this is crucial for designing hydrogels that support cellular responses associated with tissue regeneration. The study hypothesis was that dynamic hydrogels, due to their stress-relaxation properties and ability to mimic aspects of the native extracellular matrix, would differentially regulate dental pulp stem cell behavior and inflammatory responses compared with non-dynamic hydrogels. The objectives of the study are (1): Compare cellular responses and morphology by encapsulating DPSCs in D-hydrogels and ND-hydrogels (2), Characterize inflammatory modulation by quantifying and visualizing certain markers, evaluating the ability of D-hydrogels in promoting balanced immune activity.

## 2 Materials and methods

### 2.1 Hydrogel synthesis

ND-hydrogels were synthesized using Gelatin-Maleimide (Gel-Mal). Gelatin (10% w/v) was dissolved in phosphate-buffered saline (PBS) at 50 °C under stirring at 350 rpm. Maleimide functional groups were conjugated onto gelatin backbones to obtain Gel-Mal precursor. Crosslinking was achieved via thiol–maleimide reaction, enabling covalent network formation without photoinitiator or UV exposure.

D-hydrogels were prepared, leveraging host-guest interactions between Gel-Mal and Hyaluronic acid-Adamantane (HA-Ada) polymers. 1 mL of a 6% HA-Ada solution was combined with an equal molar amount of thiolated cyclodextrin (CD-SH); to ensure complete crosslinking between HA-Ada and CD-SH, optimizing hydrogel stability and mechanical properties. After thorough mixing and sonicating, a clear solution was formed through the complexation of adamantane guest molecules with host β-cyclodextrin groups on the polymer chain. Then mix the complex into the Gel-Mal solution. The resulting supramolecular complex was then incorporated into the Gel-Mal precursor prior to covalent crosslinking. The dynamic hydrogel group mechanical properties were measured using a stress-controlled rheometer with either 8-mm or 20-mm diameter parallel-plate geometry, and the gap was set to 0.5 mm.

### 2.2 Dental pulp stem cells: culture and experiments

#### 2.2.1 DPSCs culture

The DPSCs used in this study were previously isolated and characterized from extracted human teeth under institutional ethical approval and with informed donor consent. Cell characterization was previously confirmed according to established protocols (17). Cryopreserved DPSC stocks at passage 4 (P4) were thawed and expanded for use in the present study. Cells were cultured in Alpha Minimum Essential Medium (α-MEM) medium with 15% fetal bovine serum (FBS), 1% penicillin-streptomycin, and 1% L-glutamine at 37 °C. The medium was replaced every 2–3 days depending on cell density.

#### 2.2.2 Cells encapsulation in hydrogels

Sterile hydrogels were prepared and DPSCs were seeded at a density of 1 × 10^5^ cells/mL after quantification. Hydrogels were transferred to 24-well plates with 1 mL fresh medium and changed medium every 2–3 days. Cell adhesion and proliferation were monitored at 24-, 48-, and 72-hours post-seeding.

#### 2.2.3 Inflammatory marker expression

##### 2.2.3.1 Primary antibody staining

Cells were fixed with 1 mL of 10% formalin for 30 min at room temperature, followed by permeabilization with 0.1% Triton X-100 for 15 min to facilitate antibody penetration. After permeabilization, cells were blocked with 5% BSA for 1 h to reduce background noise. Primary antibodies targeting pro-inflammatory markers (IL-1RA and NF-κB) and pro-angiogenic marker (VEGF), were prepared in 5% BSA at appropriate dilutions (1:100 for IL-1RA and VEGF, and 1:200 for NF-κB). Samples were incubated with primary antibodies overnight at 4 °C on a shaker.

##### 2.2.3.2 Secondary antibody staining

After primary antibody incubation, samples were washed three times with 45 mL of distilled water and 2 drops of tween 20. Secondary antibody staining was performed using goat anti-rabbit antibodies (1:100 dilution) conjugated to fluorophores, along with phalloidin (1:200 dilution) to stain actin filaments. Samples were incubated for 2 h at room temperature.

##### 2.2.3.3 Nuclear staining

Two hours following secondary antibody staining, the secondary stain was removed, and nuclei were stained using 4′,6-diamidino-2-phenylindole (DAPI) (1:1,000 dilution, which is 1.2 µL in 2.4 mL BSA) for 30 min on shaker. Samples were washed three times with distilled water and tween 20 and stored in BSA buffer at 4 °C until imaging.

For imaging and analysis, stained samples (*n* = 4) were removed from the refrigerator and placed in round dishes for imaging. Water droplets were added as needed to maintain sample hydration during imaging. Samples were imaged using a Leica TCS SP8 confocal laser scanning microscope (Leica Microsystems, Wetzlar, Germany). Images were acquired in sequential scanning mode using 405-, 488-, 552-, and 638-nm laser lines corresponding to the fluorophores used. Final images were captured at a resolution of 2,048 × 2,048 pixels with a scan speed of 400 Hz, zoom factor of 1×, and line averaging of 3. Lower-resolution (512 × 512 pixels) live preview images were used during sample positioning and focusing before acquisition of the final high-resolution images to minimize acquisition time while maintaining image quality. Images were processed and analyzed using image analysis software to quantify cytokine expression and cellular morphology.

Regions of interest (ROIs) were manually selected based on DAPI-positive cellular areas while excluding image borders and artifacts. Fluorescence intensity was quantified using ImageJ software (National Institutes of Health, Bethesda, MD, USA). Mean fluorescence intensity was calculated within each ROI after background subtraction. To account for variations in cell density among samples, fluorescence signals for the target proteins were normalized to the corresponding DAPI fluorescence intensity within the same field. Quantitative data were expressed as relative fluorescence intensity normalized to DAPI-positive nuclei and averaged across all analyzed fields. Image processing and quantification were performed using identical thresholding and analysis parameters for all samples.

### 2.3 Macrophages: culture and gene expression profiling

#### 2.3.1 Cell culture

The macrophages used in this study were obtained from previously established and cryopreserved cell stocks derived under institutional ethical approval and informed consent. No new human subjects, tissues, or biological samples were collected specifically for the present study. Cells were cultured in Dulbecco's Modified Eagle Medium (DMEM) supplemented with 10% fetal bovine serum (FBS) and 1% penicillin/streptomycin at 37 °C. For expansion, macrophages were gently scraped from culture dishes (without trypsin, due to their adhesion mechanism) and resuspended in fresh medium. Cells were centrifuged at 300 revolutions per minute for 5 min and maintained with DMEM medium. Prior to hydrogel encapsulation, macrophages were quantified using a hemocytometer and viability was confirmed.

#### 2.3.2 Cells encapsulation in hydrogels

Sterile hydrogels were prepared then macrophages were seeded at a density of 1 × 10^5^ cells/mL after quantification. Hydrogels were transferred to 24-well plates with 1 mL fresh medium and changed medium every 2–3 days. Cell adhesion and proliferation were monitored at 24-, 48-, and 72-hours post-seeding.

#### 2.3.3 Gene expression analysis

After fixation using 1 mL of 10% formalin for 30 min, total RNA was extracted using Invitrogen [Thermo Fisher Scientific Inc., United States of America (USA)]. Samples were homogenized in TRIzol reagent (1 mL per 50 mg hydrogel) and smashed each sample manually for 3 min in tubes, then kept in ice for 30 min, and mixed with 200 µL chloroform. After centrifugation for phase separation for 15 min at 4 °C, the aqueous phase was collected in a fresh tube, and RNA precipitated with 500 µL isopropanol, then washed with 70% ethanol, air-dried, and resuspended in 20 µL nuclease-free water.

For cDNA synthesis, 1 µg total RNA was reverse transcribed using the High-Capacity cDNA Reverse Transcription Kit (Applied Biosystems™, Thermo Fisher Scientific Inc., USA) with a 5-fold diluted primer mix (4 µL RNA + 1 µL primer mix per reaction, for each group). Reactions were performed in a thermal cycler under the following conditions: 25 °C for 10 min (primer annealing), 37 °C for 120 min (reverse transcription), and 85 °C for 5 min (enzyme inactivation).

Gene expression was quantified using PowerUP™ SYBR™ Green Master Mix (Applied Biosystems™, Thermo Fisher Scientific Inc., USA) in 20 µL reaction volumes. Primer sequences for the target genes (TGFβ1, SMAD3, iNOS, MAPK, and CD80) and the reference gene (GAPDH) were added to each reaction. Each 20 µL reaction contained 2 µL of cDNA template, prepared by combining 9.5 µL of master mix with 5.5 µL of diluted primers per well. Primer pairs (0.5 µL each) were designed for each target gene involved in inflammatory responses. Amplification was performed on a QuantStudio system with the following thermocycling conditions: initial denaturation at 95 °C for 10 min, followed by 40 cycles of 95 °C for 15 s and 60 °C for 1 min. Gene expression was normalized to GAPDH and analyzed using the comparative 2^-ΔΔCt method and using the ND-hydrogel group as the calibrator. Data represent four independent biological replicates (*n* = 4), with each biological sample analyzed in technical triplicate.

### 2.4 Statistical analysis

Statistical analyses were performed using SPSS Statistics Version 29.0.2.0 (International Business Machines Corporation, Armonk, NY, USA). Normality of data distribution was assessed using the Shapiro–Wilk test. Given the small sample size (*n* = 4 per group), comparisons between dynamic and non-dynamic hydrogel groups were performed using the Mann–Whitney *U*-test. Immunofluorescence intensity data were expressed as median values and analyzed using the Mann–Whitney *U*-test. For immunofluorescence analyses, effect sizes were reported as mean differences with corresponding 95% confidence intervals (CI). Imaging quantification was performed using ImageJ software Version 2.14.0 (National Institutes of Health, USA) using a standardized image analysis protocol, and fluorescence intensity values were reported in arbitrary units (a.u.). For quantitative polymerase chain reaction (qPCR) experiments, four independent biological samples (*n* = 4) were analyzed, with each biological sample evaluated in technical triplicate. Relative gene expression was calculated using the comparative 2^-ΔΔCt method, normalized to GAPDH as the endogenous control. Fold-change values derived from the 2^-ΔΔCt analysis were reported as measures of effect size. Statistical significance was established at *p* < 0.05. Given the exploratory nature of the study and the limited number of target genes evaluated, adjustments for multiple comparisons were not applied. Data were presented in figures.

## 3 Results

### 3.1 Hydrogel synthesis

**Figure 1:**
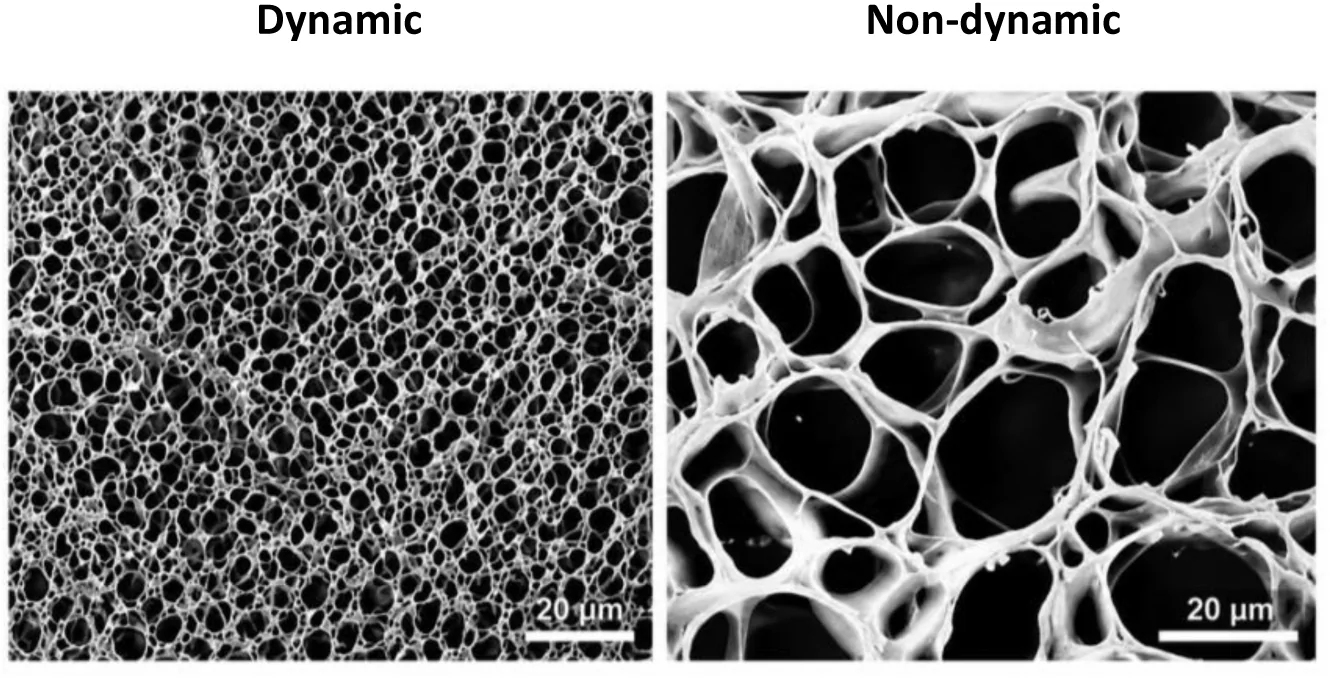
SEM images of dynamic and non-dynamic hydrogels.

A novel supramolecular dynamic hydrogel was successfully synthesized and characterized. Rheological analyses confirmed distinct mechanical behavior between the two hydrogel systems. Oscillatory frequency sweep testing demonstrated a frequency-dependent increase in the storage modulus (G′) of the dynamic hydrogel, whereas the non-dynamic hydrogel exhibited relatively stable mechanical behavior across the tested frequency range. Amplitude sweep analysis showed that the dynamic hydrogel maintained a broader linear viscoelastic region and a higher critical yield strain before structural failure, indicating greater mechanical adaptability. Furthermore, stress-relaxation testing demonstrated more rapid stress dissipation in the dynamic hydrogel than in the non-dynamic hydrogel, consistent with the reversible host–guest interactions incorporated within its network. Scanning electron microscopy (SEM) qualitatively demonstrated structural differences between the hydrogels, with the dynamic hydrogel exhibiting a denser porous architecture composed of smaller, more uniformly distributed pores than the non-dynamic hydrogel (Figure 1). Together, these findings confirm the distinct structural and viscoelastic characteristics of the dynamic hydrogel compared with the conventional non-dynamic hydrogel.

The D-hydrogel system comprised a hybrid network of Gel-Mal and HA-Ada, which forms reversible host-guest complexes with multivalent CD-SH, creating a viscoelastic matrix. In contrast, the ND-hydrogel was synthesized through irreversible crosslinking of Gel-Mal with a static HA backbone, forming a purely elastic network. This innovative design combines permanent covalent bonds with transient host-guest interactions, providing the mechanical adaptability necessary for tissue regeneration therapies.

### 3.2 Inflammatory marker expression

**Figure 2:**
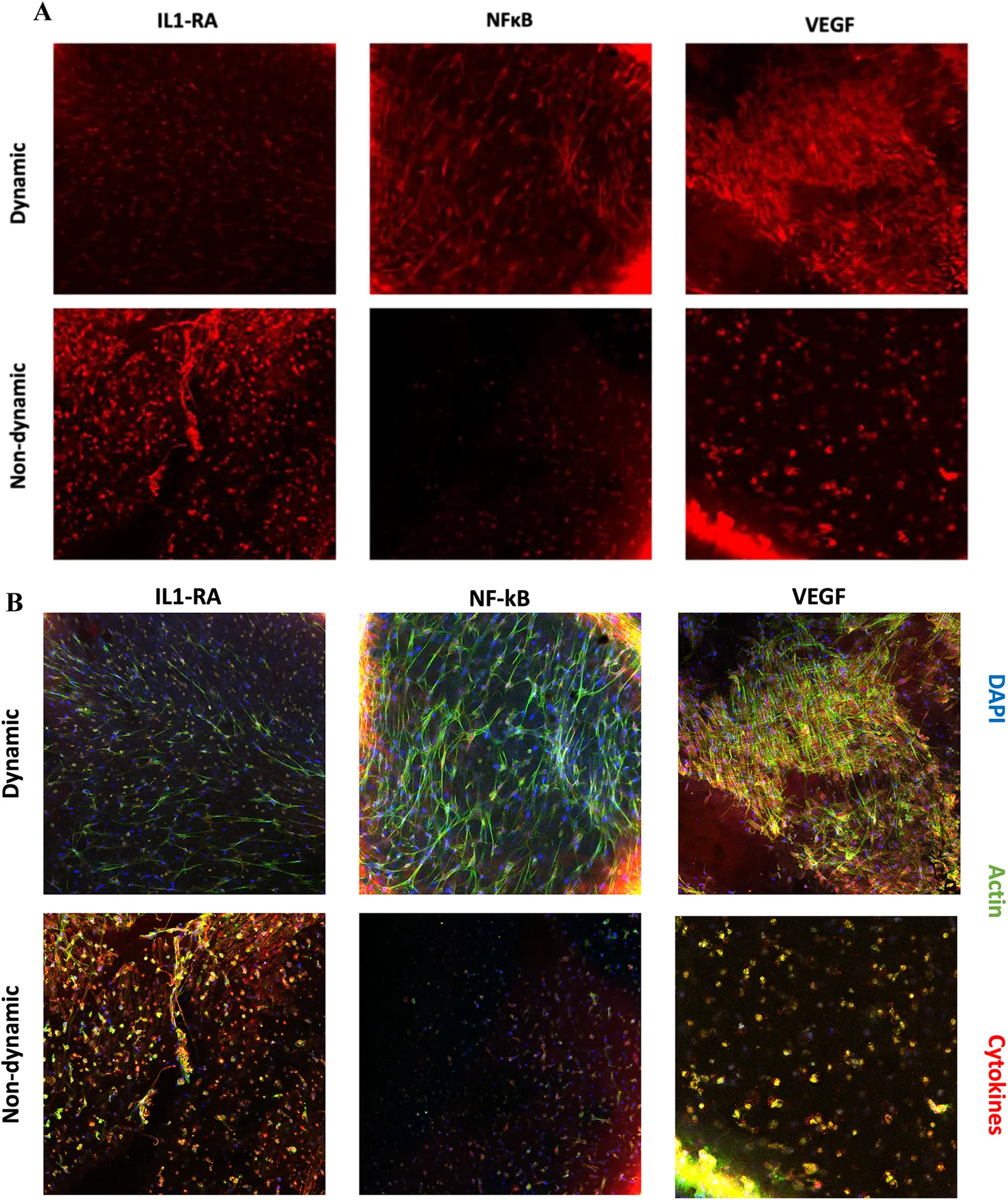
Images were acquired using a leica TCS SP8 confocal microscope with a HC PL FLUOTAR 10×/0.30 DRY objective. **(A)** Confocal images of cytokines expression show enhanced clustering in DPSCs encapsulated in D-hydrogels compared to ND-hydrogel. **(B)** Merged confocal images of cytokines expression showing DAPI, Actin, and cytokines expression.

**Figure 3:**
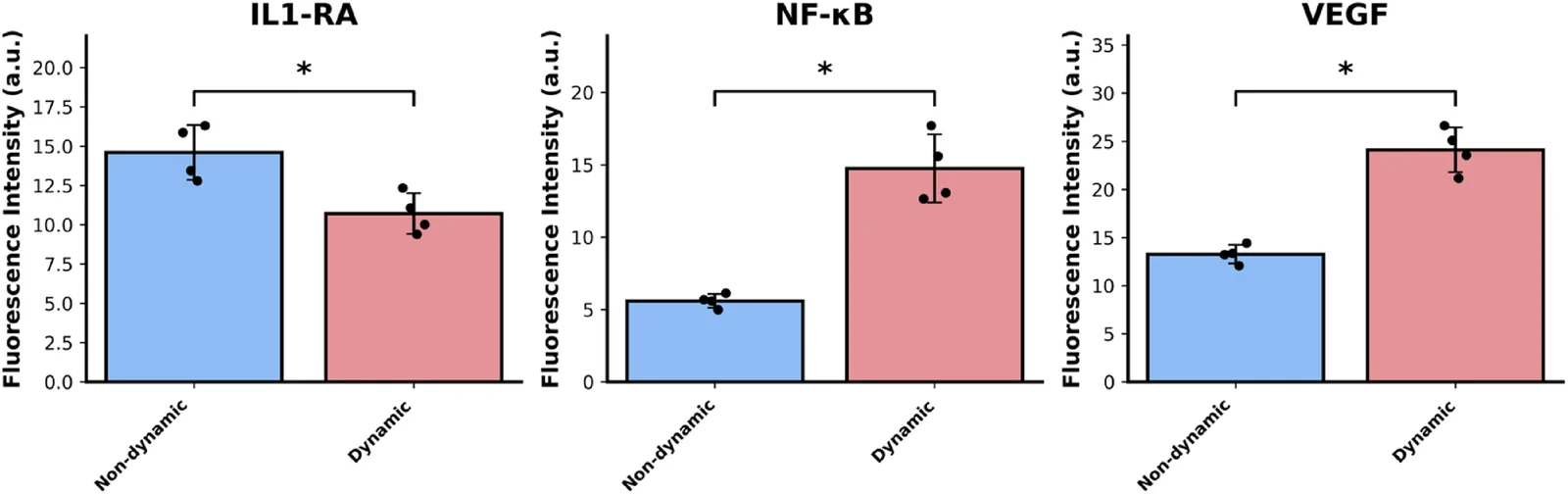
Quantification of IL-1RA, NF-κB, and VEGF fluorescence intensity in DPSCs encapsulated within dynamic and non-dynamic hydrogels. *p < 0.05.

Immunofluorescence quantification of four independent samples per group revealed mechanosensitive cytokine expression in DPSCs (Figure 2), with dynamic hydrogels (median = 10.55) showing significantly reduced IL-1RA fluorescence intensity compared to non-dynamic hydrogels (median = 14.12; U = 0, *p* = 0.0286; mean difference = −3.90, 95% CI: −6.61 to −1.18). Conversely, dynamic hydrogels significantly increased NF-κB fluorescence intensity (median = 14.33) compared to non-dynamic hydrogels (median = 5.64; U = 0, *p* = 0.029; mean difference = 9.16, 95% CI: 5.49 to 12.84). Similarly, VEGF fluorescence intensity was significantly higher in dynamic hydrogels (median = 24.34) than in non-dynamic hydrogels (median = 13.29; U = 0, *p* = 0.029; mean difference = 10.85, 95% CI: 7.35 to 14.35) (Figure 3).

### 3.3 Gene expression analysis

**Figure 4:**
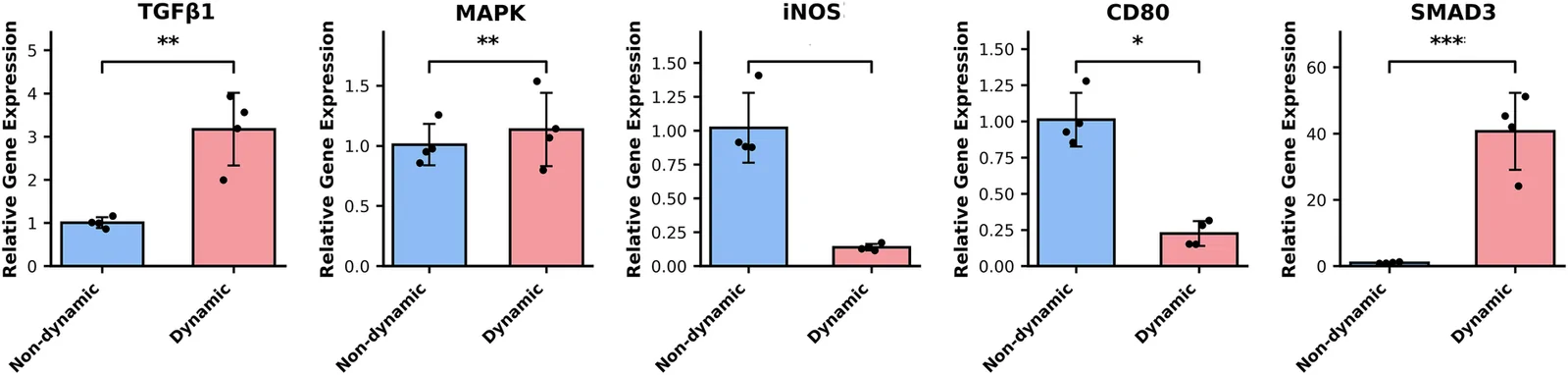
mRNA expression level of TGFβ1, SMAD3, iNOS, MAPK, CD80 in macrophages encapsulated within dynamic and non-dynamic hydrogels at day 3. Error bars represent standard deviation (SD). *p < 0.05, **p < 0.01, ****p < 0.0001.

Relative gene expression analysis demonstrated higher expression of TGFβ1, MAPK, and SMAD3 in the dynamic hydrogel group compared with the non-dynamic hydrogel group. TGFβ1 expression was significantly increased in dynamic hydrogels (fold change = 3.17, *p* = 0.0026), while SMAD3 expression was also significantly higher (fold change = 40.66, *p* < 0.001). MAPK expression showed a slight but statistically significant increase in the dynamic hydrogel group (fold change = 1.14, *p* = 0.0027). In contrast, CD80 expression was significantly lower in dynamic hydrogels compared with non-dynamic hydrogels (fold change = 0.23, *p* = 0.0134). Although iNOS expression was lower in the dynamic hydrogel group (fold change = 0.14), the difference was not statistically significant (*p* = 0.2097) (Figure 4). These findings demonstrate that D-hydrogels influence the expression of genes associated with fibrosis and inflammation compared with non-dynamic hydrogels.

## 4 Discussion

The objectives of this study were to evaluate the efficacy of D-hydrogels compared to ND-hydrogels in promoting a favorable microenvironment for cellular responses and inflammatory modulation in pulp-dentin complex regenerative procedures. DPSCs and macrophages were selected where both cell populations play critical roles during dental pulp repair. DPSCs contribute to tissue regeneration through proliferation, differentiation, and secretion of growth factors. Whereas macrophages play a central role in regulating tissue repair through their ability to coordinate inflammatory and regenerative responses (18). Therefore, evaluation of macrophage-associated markers was performed to investigate the immunomodulatory effects of dynamic hydrogel mechanics. Evaluating both cell types enabled assessment of how dynamic hydrogel mechanics may simultaneously influence cellular responses and immunomodulatory processes relevant to dental pulp regeneration.

Rheological characterization demonstrated that the dynamic hydrogel possessed distinct viscoelastic properties, including frequency-dependent mechanical behavior, an extended linear viscoelastic region, and faster stress relaxation than the non-dynamic hydrogel, consistent with its reversible host–guest crosslinking network. Consistent with these mechanical characteristics, scanning electron microscopy further demonstrated that the dynamic hydrogel exhibited a denser porous architecture with smaller pores than the non-dynamic hydrogel. Porous and interconnected architectures are considered important characteristics of regenerative scaffolds because they facilitate nutrient diffusion, cellular interactions, and tissue integration (11, 18). These results align with the scaffold requirement in regenerative endodontic therapy where it has to replicate the dynamic extracellular matrix to achieve functional tissue regeneration (16, 19).

In addition to serving as physical scaffolds, hydrogels can actively influence cellular behavior through cell–matrix interactions and immunomodulatory signaling (11). Moreover, D-hydrogels had a distinct inflammatory modulation profile, marked by NF-κB upregulation and IL-1RA suppression. While NF-κB is traditionally associated with inflammation, its transient activation is increasingly recognized as essential for initiating tissue inflammation (20). Concurrently, D-hydrogels significantly elevated VEGF expression showing the pro-angiogenic potential of D-hydrogels, a critical factor for pulp revascularization (8). These findings suggest that D-hydrogels do not simply suppress inflammatory signaling but may modulate the early inflammatory microenvironment. The observed increase in NF-κB expression with enhanced VEGF expression, may be consistent with an early inflammatory response that supports recruitment of reparative cells and initiation of angiogenesis rather than chronic inflammation. However, due to the study limitations, it requires confirmation through longitudinal studies incorporating additional inflammatory mediators. Successful regenerative therapies require modulation rather than complete elimination of inflammatory responses (18).

Interestingly, IL-1RA expression was reduced in the dynamic hydrogel group despite its established anti-inflammatory role. This finding may reflect the complex and time-dependent nature of inflammatory regulation during early tissue repair. Because the present study evaluated cytokine expression at an early time point, reduced IL-1RA expression may represent a transient response associated with initial inflammatory activation rather than a sustained pro-inflammatory phenotype. Further investigation at later time points is required to determine whether IL-1RA expression subsequently increases as tissue repair progresses. Therefore, the observed alterations in inflammatory marker expression may reflect early changes in the local inflammatory microenvironment associated with dynamic hydrogel mechanics.

Additionally, enhanced VEGF expression observed in the dynamic hydrogel group may be particularly relevant for regenerative endodontic applications because successful pulp regeneration requires establishment of a vascularized microenvironment capable of supporting cell survival and tissue repair. Previous studies have highlighted the importance of scaffold-mediated angiogenesis as a critical component of regenerative endodontic therapies (18).

The qPCR findings demonstrated that D-hydrogels induced a distinct macrophage gene expression profile compared with ND-hydrogels. Reduced CD80 expression and lower iNOS expression suggest attenuation of selected inflammatory macrophage-associated markers. Similar observations have been reported in studies demonstrating that hydrogel mechanics can influence macrophage behavior and promote regenerative immune responses rather than complete suppression of inflammation (21). In contrast, TGFβ1, SMAD3, and MAPK expression were significantly increased in the D-hydrogel group. These pathways have been associated with mechanotransduction, matrix remodeling, and regenerative cellular responses within dynamic microenvironments (19). Collectively, these findings suggest that dynamic hydrogels influence the expression of selected macrophage-associated inflammatory and tissue-remodeling genes. The reduced expression of CD80 together with the lower, although not statistically significant, iNOS expression indicates changes in selected inflammatory markers; however, these findings should not be interpreted as evidence of macrophage polarization or immune phenotype switching. Additional studies incorporating a broader panel of M1- and M2-associated markers are required to further characterize macrophage responses to dynamic hydrogels.

This study has several limitations. First, the experiments were limited to short-term *in vitro* observations and therefore cannot evaluate long-term tissue regeneration, odontogenic differentiation, or mineralized tissue formation. Second, the sample size was limited (*n* = 4 per group), which may have reduced the statistical power of the findings to detect subtle biological differences and limits the generalizability of the results. Third, inflammatory signaling was evaluated at a single early time point using a limited panel of inflammatory and fibrosis-associated markers. Although NF-κB and TGFβ/SMAD-related gene expression were examined, pathway activation was not directly confirmed through nuclear translocation, phosphorylation, or other functional signaling assays. Furthermore, the specific MAPK isoform evaluated was not investigated. Finally, while the findings demonstrate favorable cellular and immunomodulatory responses, the results should be interpreted within the limitations of an *in vitro* model, and additional *in vivo* studies are required to validate the translational potential of dynamic hydrogels for regenerative endodontic applications.

## 5 Conclusion

Within the limitations of this *in vitro* study, dynamic hydrogels demonstrated favorable cellular responses and distinct inflammatory modulation compared with non-dynamic hydrogels. These findings suggest that dynamic hydrogels may provide a biologically active microenvironment supportive of regenerative endodontic applications. Further studies evaluating cell viability, odontogenic differentiation, mineralized tissue formation, and *in vivo* performance are required before clinical translation.

## Data Availability

The original contributions presented in the study are included in the article/Supplementary Material, further inquiries can be directed to the corresponding author.
